# Predictors of early initiation of breastfeeding among Zimbabwean women: secondary analysis of ZDHS 2015

**DOI:** 10.1186/s40748-018-0097-x

**Published:** 2019-01-15

**Authors:** Fadzai Mukora-Mutseyekwa, Hilary Gunguwo, Rugare Gilson Mandigo, Paddington Mundagowa

**Affiliations:** 1 Lifestyle & Prevention Medicine Unit, Africa University Clinical Research Centre, Mutare, Zimbabwe; 2 JSI Research & Training Institute, MCHIP Project, Harare, Zimbabwe; 3 National University of Science & Technology, Bulawayo, Zimbabwe

**Keywords:** Breastfeeding, Early initiation, Predictors, Zimbabwe

## Abstract

**Background:**

The World Health Organization recommends initiation of breastfeeding within the first hour of delivery. Early initiation is beneficial for both mother and baby. Previous Zimbabwe Demographic and Health Surveys (ZDHS) have shown reduction in early initiation of breast feeding from 68% (2005/06) to 58% (2015). This study sought to investigate factors associated with early initiation of breast feeding among women aged 15–49 years in Zimbabwe.

**Methodology:**

Secondary analysis of ZDHS 2015 data was done to investigate the association between early initiation of breast feeding and maternal, provider and neonatal factors using multivariate logistic regression (*n* = 2192).

**Results:**

The majority of the study sample (78%) reported having practised early initiation of breastfeeding during their most recent delivery (preceding 24 months).Children who were put on skin to skin contact (AOR = 1.51, 95% CI 1.13–2.02) and those delivered by skilled attendants (AOR = 4.36, 95% CI 1.07–17.77) had greater odds of early initiation compared to those who were not. Other factors associated with early initiation were multiparity (AOR 1.82 95% CI 1.33–2.49) and rural residence (AOR 2.10 95% 1.12–3.93). However, having an abnormal birth weight, i.e. low birth weight (AOR 0.60 95% CI 0.36–0.99) and macrosomia (AOR = 0.42, CI 0.22–0.79) as well as delivery by caesarean section (AOR 0.1195% CI 0.06–0.19) were associated with reduced odds of early initiation.

**Conclusion:**

Early initiation of breast feeding in Zimbabwe is mainly associated with residing in the rural areas and multiparity. The 78% rate of early initiation of breastfeeding was contrary to the 58% reported in the ZDHS findings. Interventions targeting an improvement in early initiation of breastfeeding must aim at women who deliver by caesarean section, women with babies of abnormal birth weight, primi-parous women and women residing in rural areas.

## Background

The World Health Organization (WHO) recommends initiation of breastfeeding within an hour of delivery [1]. This practice is termed ‘early initiation of breastfeeding (EIBF). Early initiation is beneficial for both the mother and the baby. Benefits of this practice include exposing the newborn to colostrum, otherwise known as ‘first milk’, which is rich in protective factors [1, 2].EIBF has also been credited with facilitating bonding between mother and baby, stimulating breast milk production, reducing the incidence of post-partum haemorrhage and establishing successful and longer breastfeeding duration [3, 4]. Delaying initiation of breastfeeding is associated with a high risk of death within the first month of life [2, 5, 6].

According to UNICEF, Low to Middle Income Countries (LMICs) are disproportionately affected by suboptimal breastfeeding [7]. An analysis of DHS (Demographic Health Survey) data from 57 LMICs published in 2018 revealed that only 39% of children were breastfed within an hour of delivery (range by region of 31–60%) [8]. The 2010 Global burden of disease study revealed suboptimal breastfeeding practices to be within the top three leading contributors to disease in most countries in Sub Saharan Africa (SSA) [9].

Factors which have been shown to be associated with EIBF include maternal education, residence, household income, maternal occupation, place of birth, cultural beliefs and the availability of health facility counselling services with marked variation of influence by region [10].

Literature suggests that policy makersprioritize exclusive breastfeeding without building as much emphasis on the uptake of EIBF [11]. It has also been advanced that there has been more focus on health facility based promotion for EIBF with little effort at strategiestargeting communities [11]. In line with its National Child Survival Strategy, Zimbabwe has adopted the Baby Friendly Hospital Initiative (BFHI) as well as Infant & Young Child Feeding (IYCF) programmes aimed at promoting early initiation of breast feeding. If these initiatives are implementedproperly, they should cover health facility as well as community based interventions. However, review of serial Zimbabwe Demographic and Health Surveys (ZDHS) has revealed substantial reduction in EIBF from 68% (2005/06) to 58% (2015) [12].

**Fig. 1:**
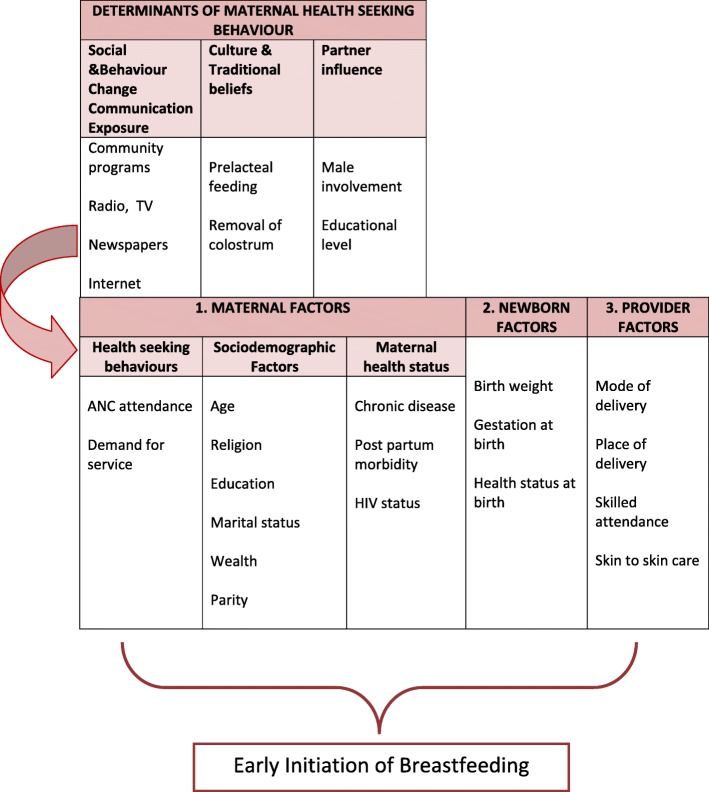
Conceptual Framework on predictors of uptake of early initiation of breastfeeding

Given this negative trend, it is important to explore the factors associated with uptake of the recommended practice of EIBF to inform and target programming efforts.This study sought to investigate the predictors of early initiation of breast feeding among women aged 15–49 years in Zimbabwe using ZDHS 2015 survey data. Specifically, the investigators set out to determine the *maternal, newborn and provider factors* associated with EIBF. Figure 1 illustrates the conceptual framework that guided the setting up of the research questions and methodology.

## Methods

### Data

**Fig. 2:**
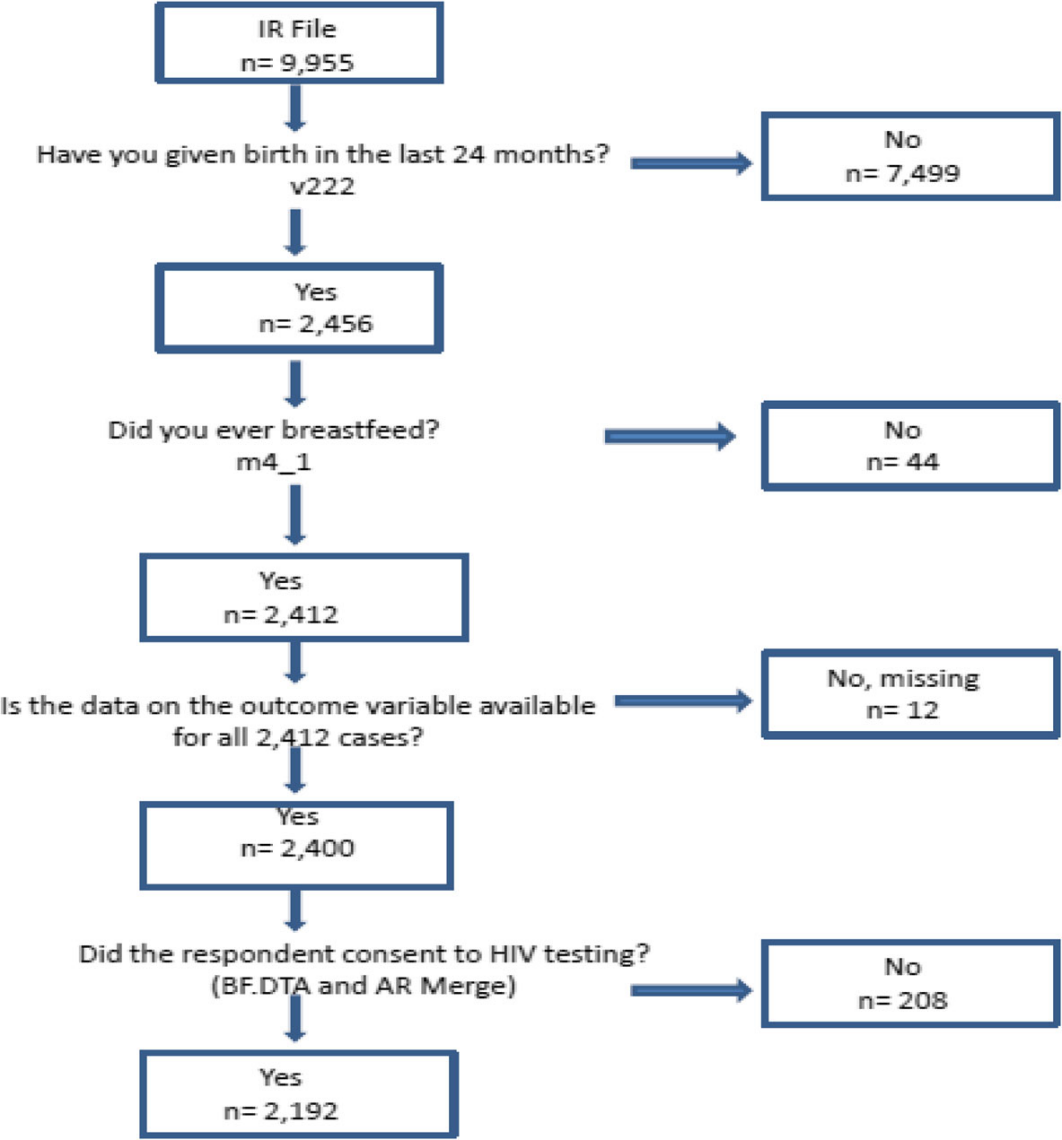
Sample selection process

Data derived from the 2015 Zimbabwe Demographic and Health Survey (ZDHS) were used for secondary analysis in this study in order to determine the predictors of early initiation of breastfeeding among Zimbabwean women. The ZDHS was conducted by the Zimbabwe National Statistical Agency on a nationally representative sample of men and women in their reproductive age. The 2015 ZDHS samples were selected using a stratified two-stage cluster design with census enumeration areas as sampling units for the 1st stage and households for the 2nd stage. All women age 15–49 who were either permanent residents of the selected households or visitors who stayed in the households the night before the survey were eligible to be interviewed. The sample size for the number of women of reproductive age in the 2015 ZDHS dataset was 9955. In this study, further selection processes were conducted as illustrated in Fig. 2 according to the following eligibility criteria:

- History of live birth over the preceding 24 month period
- History of breastfeeding
- Outcome variable data available for participant record
- Consented to HIV testing for the survey

Following these exclusions, most of the original sample of 9955 was trimmed down to the final analysis sample of *n* = 2192. This was because 75% of the sample had given birth more than 24 months prior to data collection and we sought to avoid the influence of recall bias.

### Measurement of outcome variable

The outcome variable of interest was ‘early breastfeeding initiation’, which was defined as, ‘initiation of breastfeeding within one hour after birth’.

### Independent variables

The independent variables used in this study were derived from the conceptual framework (Fig. 1) depending on availability of the information in the ZDHS dataset. They were categorized into three groups: maternal, newborn and health care provider related factors.

Maternal factors included sociodemographic indicators such as Age in years (15–19, 20–34, 35+); Residence (Rural, Urban); Religion (None, Christian, Apostolic sect, Other); Wealth quintile (Lowest, Second, Middle, Fourth, Highest); Educational level (no formal education, primary, secondary or higher education); Employment status (currently employed, not currently employed); and Parity (1, 2–4, 5 & above). The health seeking behaviour variable, Number of ANC visits was also analysed. The variable HIV status was also included in the analysis (positive, negative).

The Newborn factor included in this analysis which was available in the ZDHS dataset was Birth Weight (low birth weight, normal weight, macrosomia).

Health care provider related factors were measured using the following variables: Place of delivery (public, private);Exposure to skilled provider at delivery (Yes, No);Exposure to skin-skin contact (Yes, No), andMode of delivery (Vaginal delivery, Caesarean section).

In addition, the analysis also controlled for determinants for maternal health seeking behavior whose data was available in the DHS dataset. This included exposure to various media modalities (radio, TV, newspapers and internet) and male partner’s education level (None, Primary, Secondary, Tertiary).

### Statistical analysis

Analyses included descriptive data analyses for frequencies and proportions, chi^2^ testing for simple associations between selection of independent variables and outcome variable as well as modelling for simple and multivariate regression analyses. Prevalence and odds ratio at (95% CI: Confidence Intervals) were calculated using Stata version 14 (StataCorp, College Station, TX).

## Results

### Description of the study sample

A sample of 2192 women aged 15 to 49 underwent analysis in this study. The majority of the study sample (78%) reported having practised early initiation of breastfeeding during their most recent delivery (preceding 24 months) compared to 22% of the study population who did not practice EIBF. Table 1 shows the distribution of the study population by age, level of education and place of residence. The study sample was predominantly rural (67%), with a high literacy level (99%) which is fairly representative of these demographic features within the Zimbabwean population.

**Table 1:** Sample distribution of selected socio-demographic features (*n* = 2192)

Variable	Frequency n (%)
Residence	
Rural	1461 (67%)
Urban	731 (33%)
Age	
15–19	287 (13%)
20–34	1602 (73%)
35–49	303 (14%)
Education	
None	24 (1%)
Primary	647 (30%)
Secondary	1414 (64%)
Tertiary	107 (5%)

### Logistic regression

The results of the adjusted OR, *p*-value and 95% CI for the independent variables that emerged significant following multiple logistic regression modelling are shown in Table 2.

**Table 2:** Multivariate associations between statistically significant factors and early initiation of breastfeeding among women in Zimbabwe, 2015

	Adjusted Odds Ratio	p-value	95% confidence interval
Provider factors			
Skilled attendance at delivery	4.36	*P* < 0.01	1.07–17.77
Skin to skin intervention^a^	1.51	*P* < 0.001	1.13–2.02
Delivery by C-section	0.11	P < 0.001	0.06–0.19
Maternal factors			
Residing in rural areas	2.10	P < 0.01	1.2–3.93
Parity (Multiparity^b^)	1.82	P < 0.001	1.33–2.49
Newborn factors			
Low birth weight^c^	0.6	P < 0.01	0.36–0.99
Macrosomia^d^	0.42	P < 0.001	0.22–0.79

Results are adjusted for mother’s HIV status, parity, exposure to the internet, ANC attendance, birth weight, place of delivery, husband’s educational attainment, respondent’s level of education, exposure to radio, cell phone ownership, marital status, wealth quintile, place of ANC, ANC provider

^a^It is recommended that newborns be placed on their mother’s chest immediately after birth as part of the Baby Friendly Hospital initiative and serves to prevent neonatal hypothermia

^b^History of 2 or more children

^c^Birth weight < 2500 g

^d^Birth weight > 4000 g

## Discussion

This study aimed at investigating the predictors of early initiation of breast feeding among women aged 15–49 years in Zimbabwe using secondary data from the ZDHS 2015 survey. The majority of the study sample (78%) reported having practised EIBF during their most recent delivery (preceding 24 months) and this EIBF rate was higher than original findings from the ZDHS 2015 survey. The sample used for this analysis was confined to the preceding 24 month period in order to cater for the possibility of recall bias. The discrepancy may be explained by the increased thrust from the Ministry of Health & Child Care on improving Quality of Care during the delivery process in more recent times thus improving the practice and uptake of early initiation of breastfeeding in the period under study compared to the whole DHS sample. The findings in this sample also show a higher EIBF rate than other studiesin Chipinge, Zimbabwe (52%) and, rural Tanzania (51%) and South East Asia (25%) [13–15].

Having health workers who are skilled before, during and after the baby is delivered is vital in ensuring successful EIBF and in our study, a number of health care provider related factors emerged as determinants for the practice of early initiation of breastfeeding.

Attendance by skilled health personnel at delivery was shown to be a strong predictor for achieving EIBF among Zimbabwean women. This aligns with literature from other settings where delivery through traditional birth attendants or other non-skilled cadres was associated with delayed breastfeeding initiation [8, 13]. A qualitative study carried out in Ghana also revealed the perception from communities that health facility delivery would facilitate EIBF because the staff encouraged the practice [11].This demonstrates that most skilled health providers were trained in both teaching and skill for successful EIBF and were effectively passing it on to the mothers. However, this factor had a wider 95% Confidence Interval range which lowered its statistical significance.

We found thatwomen who delivered by caesarean section were less likely to practice EIBF compared to those who had a vaginal delivery. A secondary analysis paper of the WHO Global survey published in 2017 also showedEIBF to be significantly lower among women with complications during pregnancy and caesarean section delivery [15]. Similar findings have also been reported in other settings [14–16]. Caesarean section birth has been found to be significantly associated with higher rates of pre-lacteal feeding which militates against EIBF [15, 17]. This finding can be attributed to lengthy postoperative care which delays mother-baby contact.

The convenient and natural intervention of skin to skin contact is vital to survival of the newborns yet it is still unpopular due to separation of the mother-baby pair for routine post-delivery procedures [18].This study revealed that women who reported having been subjected to skin to skin contact with their babies immediately post-delivery had higher odds of having practiced EIBF. This finding is in line with evidence which shows that immediate post-partum skin-to-skin contact between mother and newborn facilitates EIBF and has other advantages [1, 18]. Early mother-baby contact soon after delivery enhances EIBF as well as improving the mother-baby relationship [19].

Birth weights outside of the normal ranges of 2500 to 4000 g were also found to be associated with delayed initiation of breastfeeding in this study. Mothers who delivered low birth weight babies were also shown to be less likely to initiate breastfeeding early in other studies in similar settings [13].In most settings, abnormal weight babies tend to be separated from their mothers for longer periods post-delivery as they may suffer from other morbidities requiring intervention. However, this separation then also exacerbates the situation as the babies fail to access the advantages of EIBF.

Multiparity was also found to be significantly associated with increased rates of EIBF and this is consistent with previous studies in which mothers who had three or more children had nearly twice higher odds of EIBF within one hour of birth compared to first time mothers [10, 16]. We postulated that first pregnancies tend to have a higher incidence of delivery complications which may result in separation of the mother-baby pair. However, it may also be reflective of the lack of knowledge on the importance of EIBF and therefore low demand, which then improves with serial pregnancies.

Contrary to the findings by the Tanzanian DHS which showed that urban mothers have a higher uptake of EIBF than rural mothers (62 and 45% respectively) [20], we found out that mothers residing in rural areas were more likely to practise EIBF in the Zimbabwean setting.

The ZDHS data that was used for this analysis is nationally representative and results from application of strict standardised data collection protocols administered by trained study personnel using validated questionnaires, thus rendering a strong basis for generalization and translation of findings to inform policy and practice.

However some limitations arose because of the secondary nature of the methodology. For example because secondary data was used, information on other important factors highlighted in the literature review and the conceptual framework e.g. pre-lacteal feeding, could not be made available. The DHS data did not have comprehensive information on babies who were born at home and their weight was unknown. It was also not possible to establish causality due to the cross-sectional methodology employed in this population based survey.

## Conclusions

Early initiation of breast feeding in Zimbabwe is mainly associated with residing in the rural areas and multiparity. Therefore, there is need to invest in programs to improve the practice among women living in urban settings and there is also need to strengthen support for nulliparous women on breastfeeding education during ANC, delivery and immediately post-partum e.g. pregnancy and lactation support groups. Additional focus must be given toearlier breastfeeding initiation for stable caesarean section and abnormal birth weight deliveries.

## Abbreviations

BFHI: Baby Friendly Hospital Initiative
DHS: Demographic & Health survey
EIBF: Early Initiation of Breastfeeding
HIV: Human Immunodeficiency Virus
IYCF: Infant & Young Child Feeding
LMICs: Low to Middle Income Countries
SSA: Sub Saharan Africa
WHO: World Health Organization
ZDHS: Zimbabwe Demographic & Health Survey
